# Supplementary material for: Value-Added Utilization of Citrus Peels in Improving Functional Properties and Probiotic Viability of Acidophilus-bifidus-thermophilus (ABT)-Type Synbiotic Yoghurt during Cold Storage
Source: Foods. 2022 Sep 2;11(17):2677. doi: 10.3390/foods11172677 (PMC9455927; doi:10.3390/foods11172677)
Supplement: Supplementary file 1 [file foods-11-02677-s001.zip › foods-1837184-supplementary.pdf]

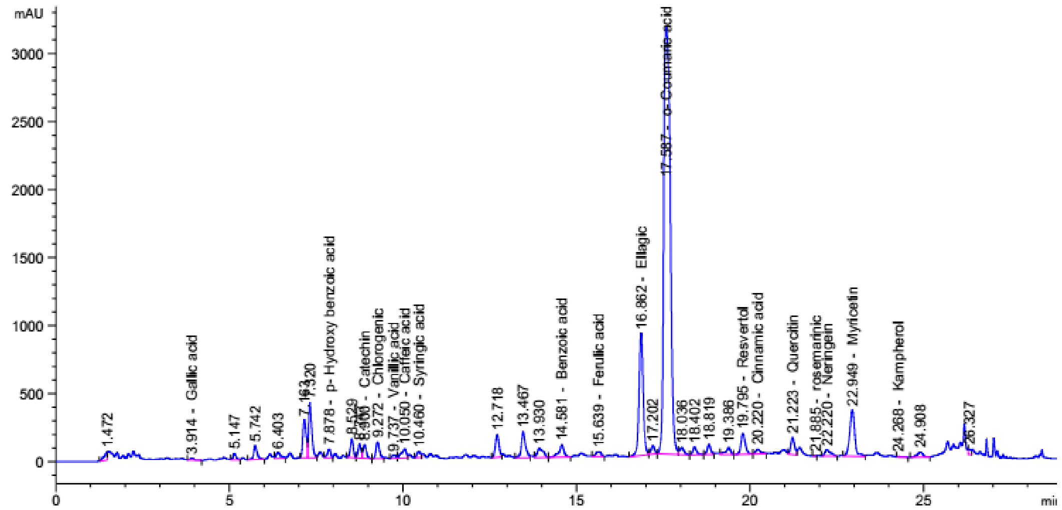

Figure S1. HPLC chromatogram of SO peel extract integrated at 284 nm

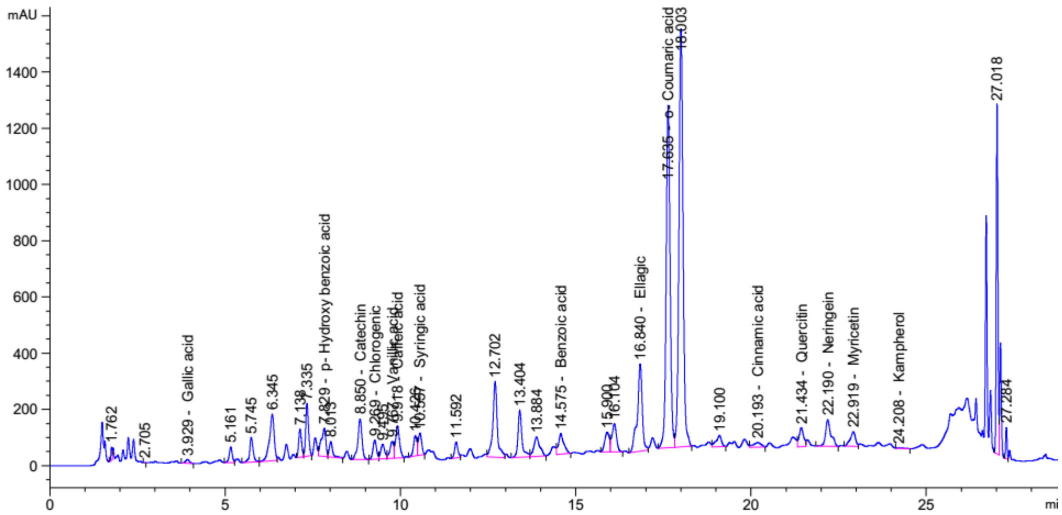

Figure S2. HPLC chromatogram of SWO peel extract integrated at 284 nm

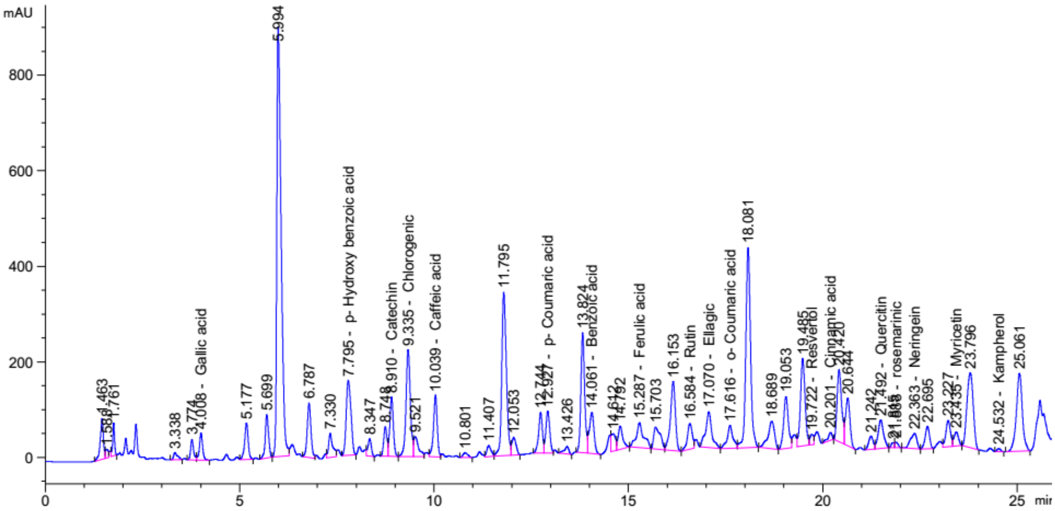

Figure S3. HPLC chromatogram of LO peel extract integrated at 284 nm

Table S1. Validation parameters for HPLC-DAD determinations of polyphenols in citrus peel extracts.

Analyte	R_t (time)	Linear equation (y=ax+b)	R^2	Linear range (mg/L)	LOD (μg/L)	LOQ (μg/L)	Intra-day precision (%)	Inter-day precision (%)
Gallic acid	3.91	y=141290x-492.8	0.9959	0.01-1	0.039	0.113	1.51	3.52
Catechin	5.40	y=100585x+818.4	0.9998	0.03-4	0.048	0.144	2.04	4.25
p-Hydroxybenzoic acid	7.88	y=30452x-1723.6	0.9951	0.005-1	0.043	0.132	1.03	3.47
Chlorogenic acid	9.27	y=49315x-825.1	0.9994	0.05-2	0.019	0.060	0.94	1.91
Vanillic acid	9.74	y=90438x-1471.8	0.9999	0.5-3	0.051	0.149	1.11	2.98
Caffeic acid	10.05	y=101252x-706.1	0.9962	0.01-1	0.010	0.033	0.85	1.54
Syringic acid	10.46	y=69438x-1095.5	0.9998	0.01-1	0.070	0.206	1.83	5.73
p-Coumaric acid	13.06	y=160581x+815.9	0.9994	0.01-1	0.040	0.123	2.61	6.91
Benzoic acid	14.58	y=58452x-2940.8	0.9999	0.01-1	0.002	0.005	1.82	5.19
Ferulic acid	15.64	y=50755x+1526.3	0.9969	0.005-1	0.049	0.168	1.06	4.55
Rutin	16.70	y=36828x+993.6	0.9995	0.03-3	0.073	0.215	1.18	3.82
Ellagic acid	16.86	y=66692x-1940.9	0.9983	0.02-2	0.082	0.239	0.9	1.92
o-Coumaric acid	17.59	y=70895x-1893.2	0.9993	0.5-5	0.091	0.028	0.42	1.73
Resveratrol	19.80	y=109410x+1023.6	0.9964	0.05-1	0.068	0.186	2.04	5.28
Cinnamic acid	20.22	y=110452x-982.7	0.9989	0.02-1	0.053	0.157	1.15	3.59
Quercetin	21.22	y=36327x-3345.8	0.9991	0.02-1	0.062	0.173	2.34	7.13
Rosemarinic acid	21.89	y=91782x+8619.3	0.9999	0.02-1	0.079	0.213	0.85	2.81
Naringin	22.22	y=44694x-3749.8	0.9995	0.02-1	0.036	0.072	1.24	4.92
Myricetin	22.95	y=51396x-5721.7	0.9993	0.5-5	0.035	0.106	0.83	4.18
Kaempferol	24.27	y=30451x-1723.6	0.9976	0.02-1	0.054	0.152	2.36	5.06

Table S2. Profiles of polyphenolic compounds (μg/g milk) in ABT synbiotic yoghurt with citrus peel addition.

Compounds		ABT fermented milk with different citrus peels		
		SO peel	SWO peel	LO peel
Phenolic acids				
1	*o*-Coumaric acid	5.15 ± 0.12	1.24 ± 0.05	0.07 ± 0.00
2	Benzoic acid	4.72 ± 0.05	4.13 ± 0.07	3.79 ± 0.10
3	Ellagic acid	2.96 ± 0.05	1.02 ± 0.05	0.31 ± 0.02
4	*p*-Hydroxybenzoic acid	0.42 ± 0.01	0.81 ± 0.06	1.21 ± 0.03
5	Chlorogenic acid	0.28 ± 0.01	0.12 ± 0.01	0.48 ± 0.06
6	Caffeic acid	0.11 ± 0.01	0.15 ± 0.01	0.14 ± 0.01
7	Cinnamic acid	0.07 ± 0.01	0.05 ± 0.01	0.01 ± 0.00
8	Gallic acid	0.03 ± 0.00	0.03 ± 0.00	0.08 ± 0.00
9	Vanillic acid	0.07 ± 0.01	0.24 ± 0.01	n.d.
10	Syringic acid	0.11 ± 0.00	0.15 ± 0.02	n.d.
11	Ferulic acid	0.13 ± 0.01	n.d.	0.12 ± 0.01
12	Rosemarinic acid	0.08 ± 0.01	n.d.	0.32 ± 0.01
13	*p*-Coumaric acid	n.d.	n.d.	0.09 ± 0.01
Stilbenes				
1	Resveratrol	3.79 ± 0.04	n.d.	0.48 ± 0.01
Flavonoids				
1	Myricetin	10.17 ± 0.19	1.64 ± 0.05	0.63 ± 0.03
2	Quercetin	2.85 ± 0.06	1.85 ± 0.04	1.59 ± 0.08
3	Naringin	1.83 ± 0.05	3.29 ± 0.07	1.08 ± 0.02
4	Kaempferol	0.02 ± 0.01	0.07 ± 0.01	0.08 ± 0.01
5	Catechin	0.15 ± 0.01	0.21 ± 0.01	0.11 ± 0.01
6	Rutin	n.d.	n.d.	0.81 ± 0.03
